# Data on the occurrence of a Brass texture and elastic anisotropy in laser blown powder processed superalloy IN718

**DOI:** 10.1016/j.dib.2021.107570

**Published:** 2021-11-14

**Authors:** J.F.S. Markanday, M.A. Carpenter, R.P. Thompson, S.E. Rhodes, C.P. Heason, H.J. Stone

**Affiliations:** a Department of Materials Science & Metallurgy, University of Cambridge, 27 Charles Babbage Road, Cambridge CB3 0FS, UK; b Department of Earth Sciences, University of Cambridge, Downing Street, Cambridge CB2 3EQ, UK; c Rolls-Royce plc, PO Box 31, Derby DE24 8BJ, UK

**Keywords:** Additive manufacturing, Nickel, Superalloys, Anisotropy, Resonant ultrasound spectroscopy

## Abstract

The additive manufacturing (AM) of components through laser-blown-powder directed-energy-deposition (LBP-DED) is highly applicable to the repair of aerospace components. Fabrication of superalloys with this technique, as with other AM methods, often encounters complications that include the formation of undesired phases, irregular microstructure and texture leading to anisotropic elastic properties. Heat treatments and other post-processing techniques can be used to mitigate these issues. The collected data demonstrates the effects of different heat treatment protocols on the microstructure, elastic properties, and hardness of LBP-DED IN718. In this study eight different heat treatment were used to investigate the effects of treatment time and temperature. The microstructure was investigated through SEM, with XRD and EDX used for phase analysis. The texture was characterised using SEM coupled with EBSD and the elastic properties were determined from resonant ultrasound spectroscopy.

## Specifications Table

**Table:** 

Subject area	Additive Manufacturing
More specific subject area	Characterization of the texture and elastic anisotropy of LBP-DED IN 718
Type of data	Figure and Table
How data was acquired	A phase analysis was carried out through X-ray diffraction (XRD), performed using a Bruker D8 X-ray diffractometer and scanning electron microscopy coupled with energy dispersive X-ray spectroscopy (SEM-EDX) using a Zeiss Gemini SEM 450 equipped with an Oxford Instruments X-MaxN 50 detector. Microstructure and texture analyses were performed with a Zeiss Gemini SEM 450 equipped with an Oxford Instruments Symmetry electron backscattered diffraction (EBSD) detector. Elastic properties were calculated using elastic constants determined from resonant ultrasound spectroscopy (RUS).
Data format	Raw
Parameters for Data Collection	X-ray diffraction: angular range (2θ) of 20° to 115° was chosen with a step size of 0.05° and a dwell time of 2.8 s. For higher resolution 5 s was used. RUS spectra were collected in the frequency range of 100–1200 kHz. EBSD orientation maps acquired with a step size of 2 µm from areas of 3 mm^2^; grain boundary angles were limited to >5°. For the contouring of the cubic pole figures: a cluster size of 5°, a half width of 15° and a mud of 6.
Description for Data Collection	Electro-discharge machining (EDM) was used to prepare parallelepiped samples for analysis from the LBP-DED builds. The parallelepiped samples were subjected to different heat treatments protocols. The anisotropies of the As-DED and heat-treated (HT) samples were calculated using elastic constants determined using RUS. A detailed explanation of the RUS technique has been given by Migliori and Sarrao [1].
Data source location	University of Cambridge, Department of Materials Science and Metallurgy
Data accessibility	Data is with this article for all figures excluding Fig. 3. Data for Fig. 3 is located in the following repository: Repository name: Apollo Data identification number: Samples As-DED to Sample F Direct URL to data: https://doi.org/10.17863/CAM.75930
Related Research Article	J. F. S. Markanday, M. A. Carpenter, R. P. Thompson, S. E. Rhodes, C. P. Heason, H. J. Stone, Occurrence of a brass texture and elastic anisotropy in laser blown powder processed superalloy IN718, Mat. Sci. & Eng. A, 2021. 825: p. 141,781 https://doi.org/10.1016/j.msea.2021.141781

## Value of the Data

• These collected data provide useful insight to researchers on how the texture and elastic anisotropy of an additively manufactured superalloy evolves under different heat treatment protocols.
• A Brass texture component, {011} 〈211〉, is observed to occur in LBP-DED IN718. Identification of this texture might be useful for aerospace design considerations of additively manufactured Ni-based superalloy components.
• The *in-situ* resonant ultrasound spectroscopy data show the evolution of elastic constants with time and temperature. This data might be useful in the optimisation of post-processing protocols for AM superalloys components.

## 1 Data Description

Detailed sample information including the dimensions and masses of the EDM parallelepiped samples have been provided in Table 1. Details of the heat treatment protocols applied to these parallelepiped samples have also been given in Table 2. A microstructural and phase analysis was completed using the SEM-EDX and XRD techniques. The XRD analysis of the AM IN718 in the As-DED and heat-treatment F condition are presented. SEM images of the AM IN718 parallelepiped samples following the different heat treatment protocols have been presented. The raw data for these analyses can be found in the supplementary data. SEM-EBSD analysis of the samples in different heat-treatment conditions have been given. The raw data for the EBSD analysis has been provided in the open access Apollo repository with the link in the Specification Table. These raw data are to be used in the Bruker AzTec software and the project files have been provided alongside the raw data files. Using the RUS spectra of the parallelepiped samples the elastic stiffness coefficients, anisotropy factors and acoustic loss data were determined. The raw data for the acoustic loss analysis can be found in the supplementary data. High temperature RUS analysis was completed on an IN718 parallelepiped sample.

**Table 1:** Dimensions and masses of the EDM prepared LBP-DED IN718 parallelepiped samples.

*Sample*	1	2	3	4	5	6	7
Dimension X (mm)	3.913	3.554	3.758	3.147	4.255	3.657	4.007
Dimension Y (mm)	2.846	2.588	1.758	2.254	2.144	0.2193	2.083
Dimension Z (mm)	4.907	4.374	4.907	3.653	3.585	0.441	3.568
Mass (g)	0.447	0.326	0.263	0.210	0.266	0.288	0.288

**Table 2:** Heat Treatment protocols applied to the LBP-DED parallelepiped samples, this table is ‘as-presented’ in the original study [2] for clarity purposes.

Heat Treatment	As-DED	A	B	C	D	E	F	G
Sample(s)	All	1/3	2	3	4	5	6	7
Temperature/°C	–	720/620	1080	1090	1100	1100	1100	1100
Time/min	–	480 / 480	10	10	10	30	60	120
Cooling	–	Air	Air	Air	Air	Air	Air	Air
Notes	Machined Condition	Two stage ppt treatment	ReX Start	ReX Start	ReX Start	Partial ReX	Partial ReX	Partial ReX

## 2 Experimental Design, Materials and Methods

Monolithic IN718 builds were manufactured using the LBP-DED technique. For analysis, parallelepiped samples were prepared from these builds using EDM. The dimensions and masses of these samples are given in Table 1.

These parallelepiped samples were subjected to different heat treatments protocols prior to analysis. The heat treatment protocols applied to the individual parallelepiped samples are presented in Table 2.

**Fig. 1:**
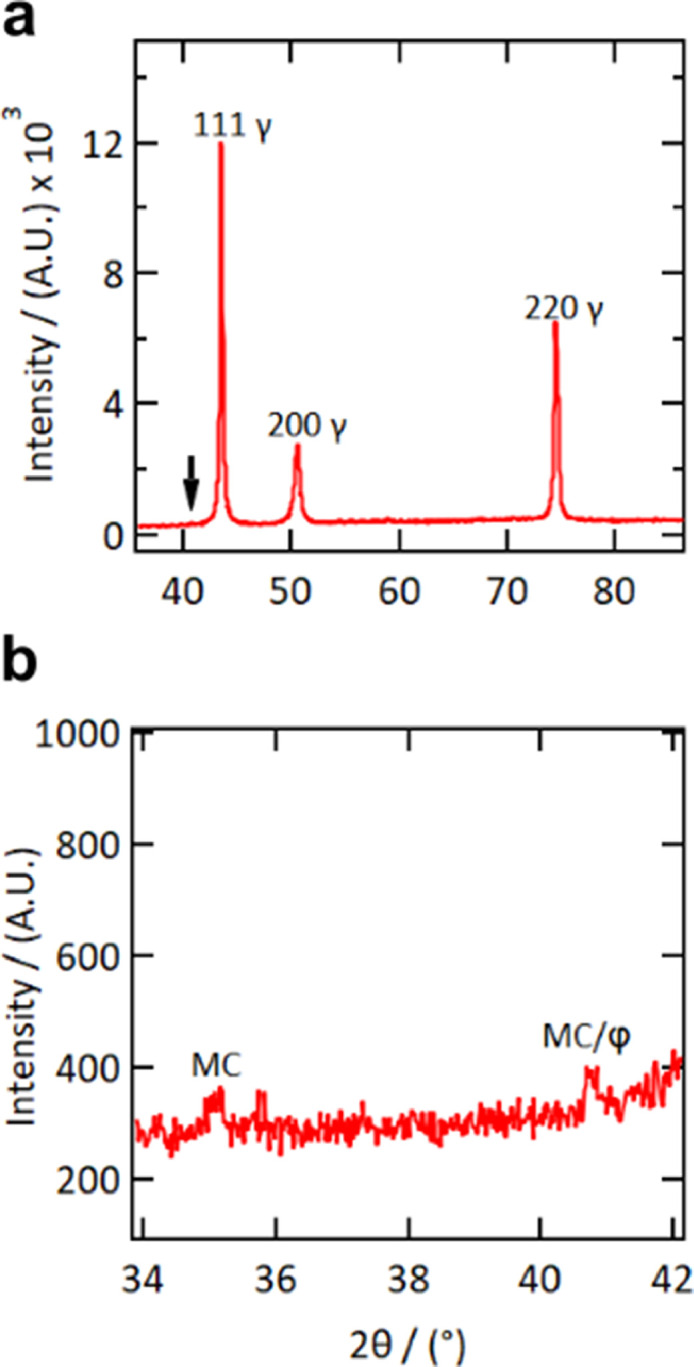
XRD patterns for LBP-DED IN718 parallelepiped Sample 6, the reflections from the gamma phase and MC type carbide and Laves phase are labelled. (a) Heat treatment F condition, a magnified view of the XRD pattern in the vicinity of the arrow shown in (b). The raw data for this figure can be found in the supplementary data.

Phase and microstructural analysis of the parallelepiped samples was carried out following the heat treatment protocols. XRD analysis of the LBP-DED IN718 was performed using a Bruker D8 X-ray diffractometer equipped with a Cu-Kα source operated at 40 kV and 40 mA. The XRD patterns for Sample 6 in the As-DED and heat-treated condition are presented in Fig. 1. The γ phase peaks can be readily identified from these patterns and are indexed in Fig. 1a. However, the superlattice reflections from the γ′ and the γ″ phases were not readily discernible. Peaks at positions 35°, 37.5° and 41° 2Θ were identified in the magnified XRD patterns of the As-DED samples in the original study [2]. These peaks indicate the presence of MC type carbides and the Laves phase in the microstructure. It must be noted that the peak at position 41° is attributed to both the MC type carbide and Laves phase. These two peaks were retained after the heat treatment at high temperature suggesting that they are associated with the MC carbide phase. There was no detection of the 202 reflection of the Laves phase, which normally occurs at 45° 2Θ. This observation indicates that the Laves phase has been dissolved following heat-treatment F.

**Fig. 2:**
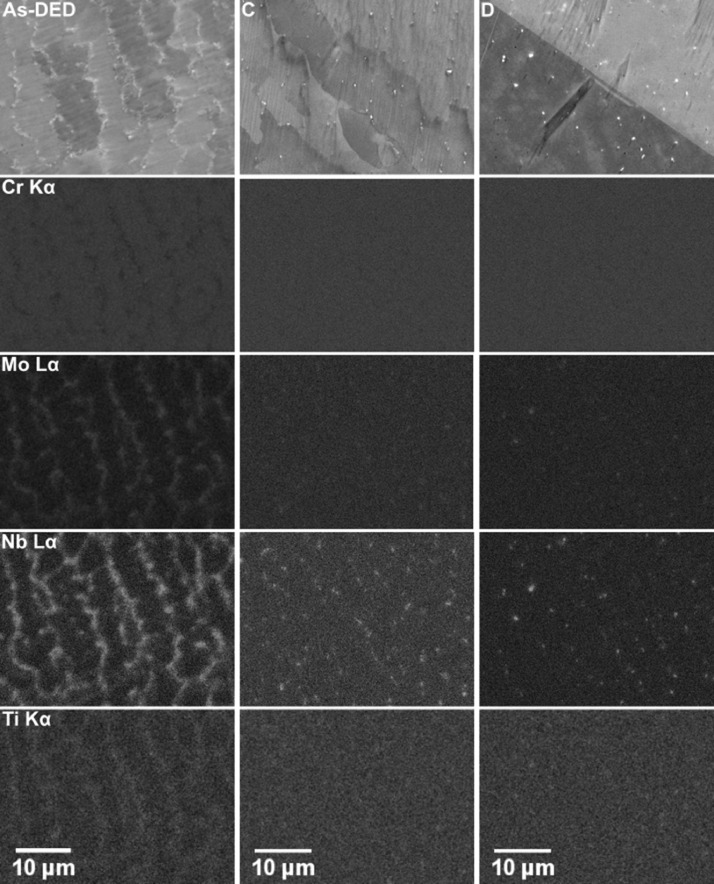
SEM analysis LBP-DED IN718 samples in different processing conditions: As-DED and heat treatments C and D. For each sample, a backscattered electron image is shown at the top, below which are the Cr, Mo, Nb, and Ti elemental distribution maps obtained by SEM-EDX. The raw data for this figure can be found in the supplementary data.

**Fig. 3:**
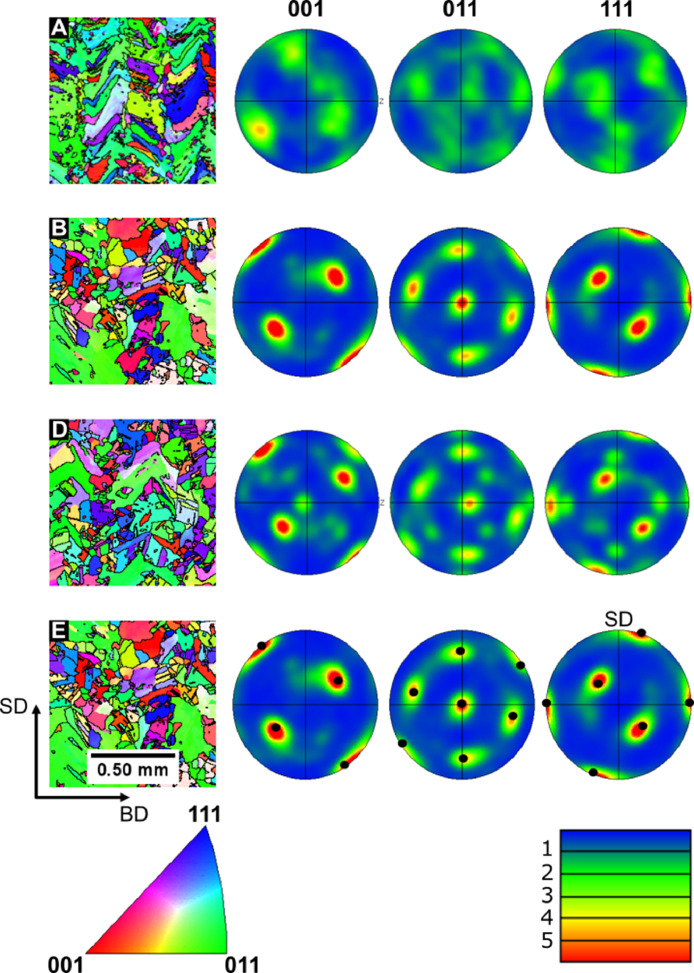
Inverse pole figure maps with respect to the build direction (BD) and scanning direction (SD) for LBP-DED RUS parallelepiped samples in the heat-treated A, B, D and E Conditions. Right–Corresponding {001}, {011} and {111} pole figures in the BD plane. The idealised spots for the {011} 〈211〉 Brass component are identified by black spots. The raw data for this figure has been published in open Apollo repository, see Ref. [3].

SEM and EDX analysis were performed using a Zeiss Gemini SEM 450 operated at 20 kV and equipped with an Oxford Instruments X-MaxN 50 detector. The SEM results obtained from the parallelepiped samples in the As-DED, C and D heat-treatment conditions are given in Fig. 2. For the As-DED condition, a cored dendritic microstructure was observed. There has been significant segregation of the refractory elements to interdendritic regions. Images for samples in the A, B and F heat-treatment conditions can be found in the original study [2]. Numerous inter-dendritic precipitates were identified from the EDX analysis, these phases were primarily rich in Mo, Nb and Ti. Several precipitates were also identified that had a high concentration of Cr. It is suspected that these phases were Laves or carbides due to the enrichment of the aforementioned elements.

Following high-temperature heat treatments the inter-dendritic structures were observed to have been dissolved and the elemental segregation reduced. The fine precipitates rich in Nb and Mo that remained following high-temperature heat treatments are suspected to be carbides, this is supported by the XRD results.

A texture analysis of the LBP-DED IN 718 parallelepiped samples was performed using a Zeiss Gemini SEM 450 equipped with an Oxford Instruments Symmetry EBSD detector. The EBSD results for the samples in the different heat treatment conditions are presented in Fig. 3. Additional results for samples in other conditions can be found in the original study [2]. EBSD orientation maps were acquired with a step size of 2 µm from areas of 3 mm^2^; grain or cell boundaries boundary angles were limited to >5° for EBSD grain boundary maps. The following parameters were used for the contouring of the cubic pole figures: a cluster size of 5°, a half width of 15° and a maximum intensity for the multiples of uniform density (mud) of 6 mud. A Brass textural component was identified in the EBSD analysis, this component was shown to be enhanced with heat treatment duration at higher temperatures. In the A condition the texture component is clearly weaker. There is a strengthening of this texture during the partial recrystallisation heat treatments. This was hypothesised to be a result of the carbides restricting growth of grains during the recrystallisation processes.

**Table 3:** Elastic constants and properties for the LBP-DED IN718 parallelepiped samples in their respective heat treatment (HT) conditions. The sample has been labelled in brackets beneath the heat treatment (HT) condition. The elastic constants were calculated with sample dimensions for the RD, TD and BD representing the x (1), y (2) and z (3) axes, respectively.

										B	E	G	RMS
HT (Sample)	Stiffness Coefficients (GPa)									(GPa)	(GPa)	(GPa)	(%)
	c_11_	c_22_	c_33_	c_12_	c_13_	c_23_	c_44_	c_55_	c_66_				
As-DED (1)	314 ± 2	296±1	285±2	115±2	118±3	123±2	81±0.03	71±0.01	67±0.1	178	207	79	0.37

B (2)	301±1	254± 3	282±2	109±6	114±3	118±4	76±0.05	66±0.02	67±0.01	168	194	74	0.54

D (4)	316±1	313±1	287±1	146±1	136±1	143±2	77±0.01	69±0.01	69±0.01	196	201	76	0.48

E (5)	291±3	282±4	290±1	117±2	127±2	126±3	80±0.03	74±0.02	72±0.14	178	204	78	0.62

**Table 4:** Anisotropy coefficients A_100_, A_010_ and A_001_ for the LBP-DED IN718 parallelepiped samples in their respective heat treatment (HT) conditions.

HT (Sample)	A_100_	A_010_	A_001_
As-DED (1)	0.95 ± 0.02	0.78 ± 0.02	0.79 ± 0.02
As-DED (3)	0.93 ± 0.02	0.79 ± 0.02	0.71 ± 0.02
A (3)	0.94 ± 0.02	0.79 ± 0.02	0.72 ± 0.02
B (2)	1.00 ± 0.03	0.73 ± 0.02	0.80 ± 0.02
C (3)	0.93 ± 0.02	0.80 ± 0.02	0.76 ± 0.02
D (4)	0.98 ± 0.02	0.83 ± 0.02	0.82 ± 0.02
E (5)	1.00 ± 0.02	0.90 ± 0.02	0.85 ± 0.02
F (6)	0.97 ± 0.02	0.93 ± 0.02	0.86 ± 0.02
G (7)	1.00 ± 0.03	0.91 ± 0.02	0.93 ± 0.02

**Fig. 4:**
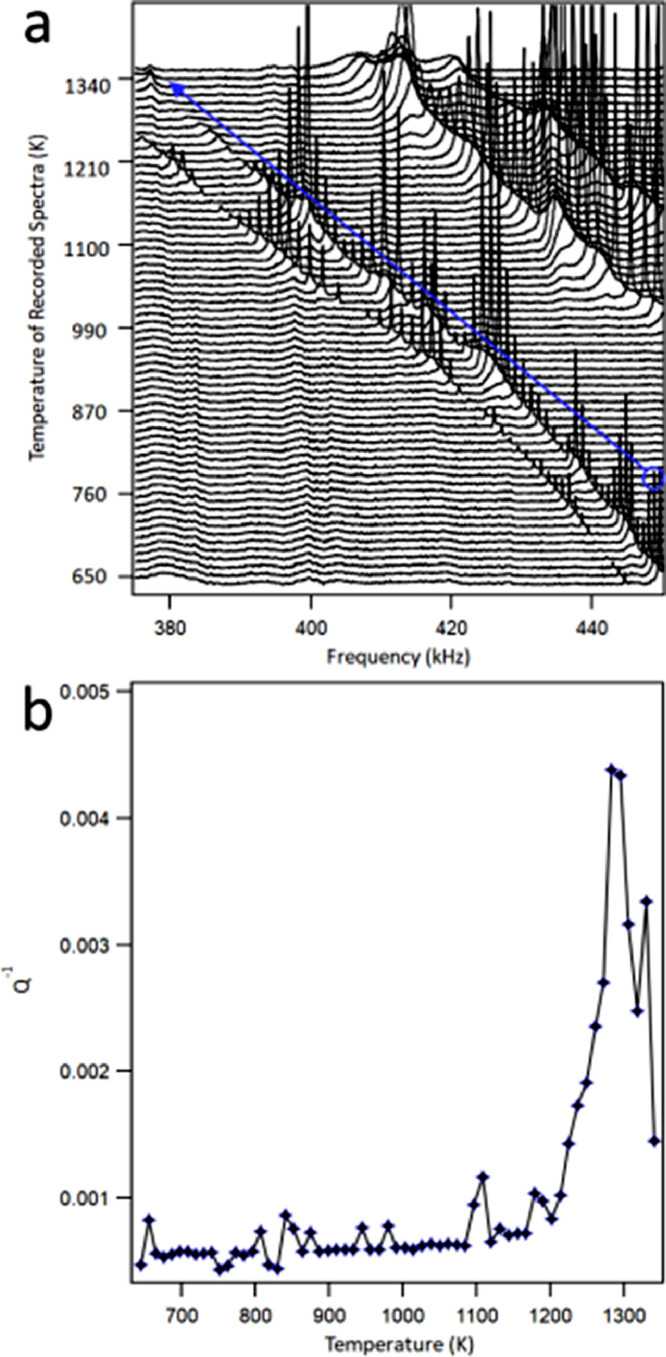
(a) - High temperature RUS spectra of an LBP-DED parallelepiped sample collected during heating, a circle (blue) is shown above the first resonant peaks in the series of interest. An arrow (blue) has been added as a guide to the eye to allow tracking of the peaks through the temperature range. (b) – Acoustic loss plotted against temperature for each resonant peak in the series of interest. The raw data for this figure can be found in the supplementary data.

The results from the RUS analysis are presented in Table 3, additional results for samples in different heat-treatment conditions can be found in the original study [2]. RUS spectra were collected at room temperature in the frequency range of 100–1200 kHz, with 50,000 data points in each spectrum. The experimental methods used for the present study are described by McKnight et al. [4,5]. For each sample, five spectra were collected with the samples mounted in a different orientation during collection so as to ensure that all the resonances in this frequency range were observed. Analysis of the RUS spectra were completed in the Wavemetrics IGOR Pro software package; with individual resonance peak frequencies identified using an asymmetric Lorentzian function. The open-source rectangular parallelepiped resonances (RPR) code [1] was used to analyse the resonant frequencies from each sample to calculate the elastic stiffness coefficients. Using the RUS data, the elastic stiffness coefficients and elastic moduli were calculated for each of the LBP-DED IN718 parallelepiped samples following the specified heat treatment protocols. The associated uncertainty for the calculated elastic moduli was consistently below 2.5%, with the shear components exhibiting the lowest uncertainty due to the resonant modes of the samples having a predominantly shear character. It is quite clear from the elastic constants that the samples do not display isotropic elastic properties. Indeed, by closer inspection the samples display an elastic symmetry closer to hexagonal, which strengthens with the higher temperature heat-treatments.

The elastic anisotropy of the samples in the cubic shear-planes was quantified into three terms A_100_, A_010_ and A_001_ (Eqs. 1–3), where an isotropic single crystal would yield values of 1 for all terms. The calculation of these coefficients was derived from the work of Ravindran et al. [6]. The present study has applied the approach to RUS data from the textured polycrystalline samples. This is in contrast to the data from a single crystal, as used in the original study. The anisotropy coefficients for the samples are given in Table 4. The anisotropy is clearly reduced with heat-treatment temperature and duration. However, there is still residual elastic anisotropy along the cubic planes.

(1) $$A_{100}=\frac{4C_{44}}{C_{22}\;+\;C_{33}\;-\;C_{23}}$$

(2) $$A_{010}=\frac{4C_{55}}{C_{11}\;+\;C_{33}-\;C_{13}}$$

(3) $$A_{001}=\frac{4C_{66}}{C_{11}\;+\;C_{22}\;-\;C_{12}}$$

High-temperature RUS was used to monitor the change in the elastic constants with temperature. A single peak from the RUS spectra was chosen for analysis throughout the recorded temperature range. From the resonant peaks the acoustic loss was calculated to probe for thermally activated processes. Sharp increases in the acoustic loss can indicate the start of processes such as the diffusion of elements and the dissolution of precipitates [7]. The acoustic loss of a single series of peaks has been calculated for an LBP-DED IN718 parallelepiped sample, and the results have been provided in Fig. 4.

The acoustic loss data can be used to probe for the thermally activated processes with the LBP-DED IN718 samples. Such processes might include the dissolution of precipitates at high temperatures. The event around 1100 K in Fig. 4b is attributed to the formation of the γ″. The event near 1200 K is attributed to the dissolution of phases prior to melting of the matrix.

## Ethics Statement

No data was collected on human or animal subjects. In addition, no data was collected through social media platforms. All data collected complies with the ethical guidelines of the publisher.

## CRediT authorship contribution statement

**J.F.S. Markanday:** Conceptualization, Validation, Formal analysis, Investigation, Writing – original draft, Visualization. **M.A. Carpenter:** Methodology, Formal analysis, Resources, Investigation. **R.P. Thompson:** Formal analysis, Writing – review & editing. **S.E. Rhodes:** Investigation. **C.P. Heason:** Resources, Supervision, Funding acquisition. **H.J. Stone:** Conceptualization, Writing – review & editing, Supervision, Funding acquisition.

## Declaration of Competing Interest

The authors declare the following financial interests/personal relationships which may be considered as potential competing interests: This work was funded by the EPSRC (through and iCase studentship) and by Rolls-Royce plc.

## Appendix Supplementary materials

Image, application 1

Image, application 2

Image, application 3
