# Supplementary figures and images for: Data on the occurrence of a Brass texture and elastic anisotropy in laser blown powder processed superalloy IN718
Source: Data Brief. 2021 Nov 14;39:107570. doi: 10.1016/j.dib.2021.107570 (PMC8605062; doi:10.1016/j.dib.2021.107570)

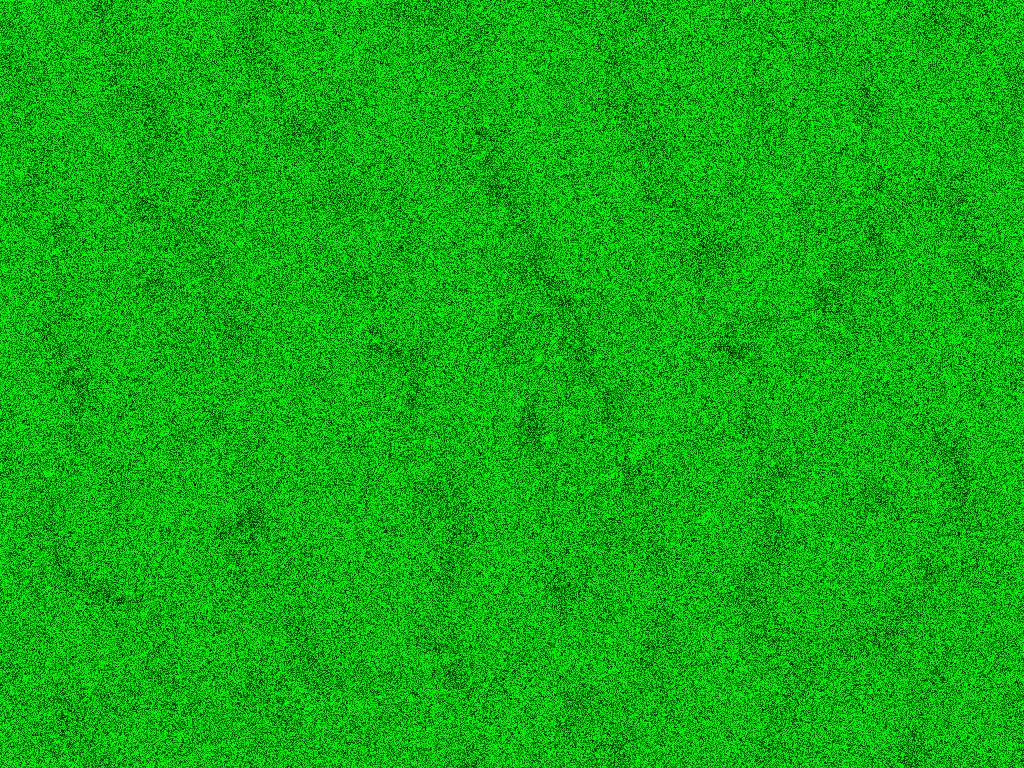

Supplement: Supplementary file 2 [file mmc2.zip › Figure 2 Raw TIFs - Markanday Data-in-Brief/As-DED/Cr K_alpha_1 Map As-DED.tif]

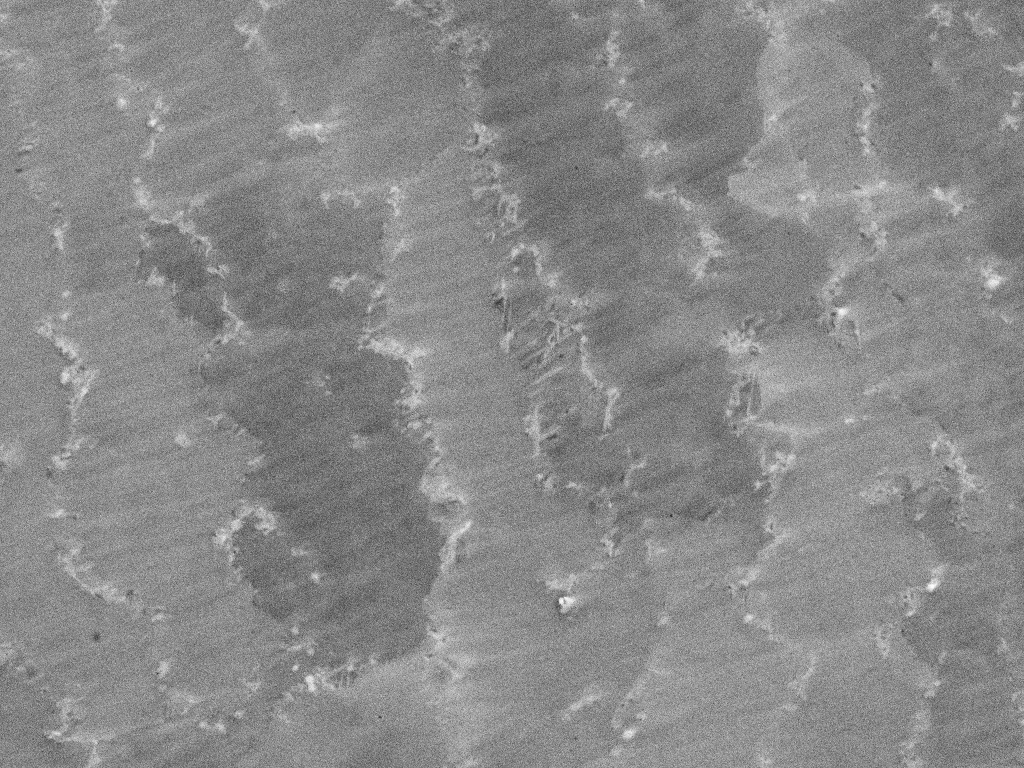

Supplement: Supplementary file 2 [file mmc2.zip › Figure 2 Raw TIFs - Markanday Data-in-Brief/As-DED/Electron Image As-DED.tif]

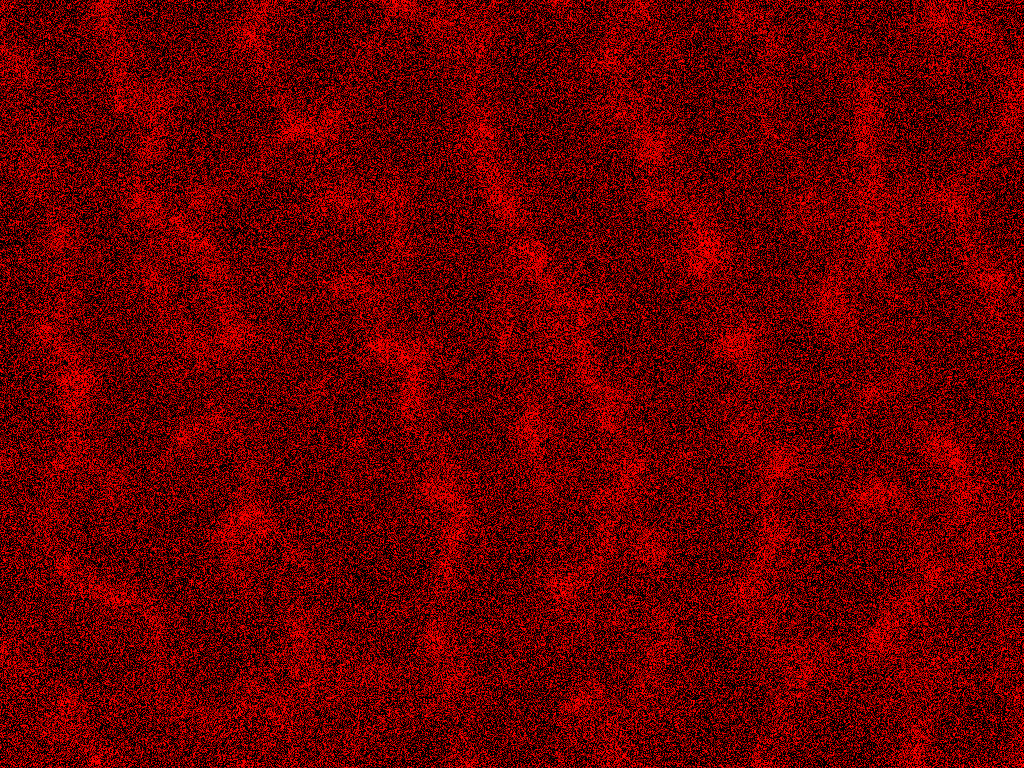

Supplement: Supplementary file 2 [file mmc2.zip › Figure 2 Raw TIFs - Markanday Data-in-Brief/As-DED/Mo L_alpha_1 Map As-DED.tif]

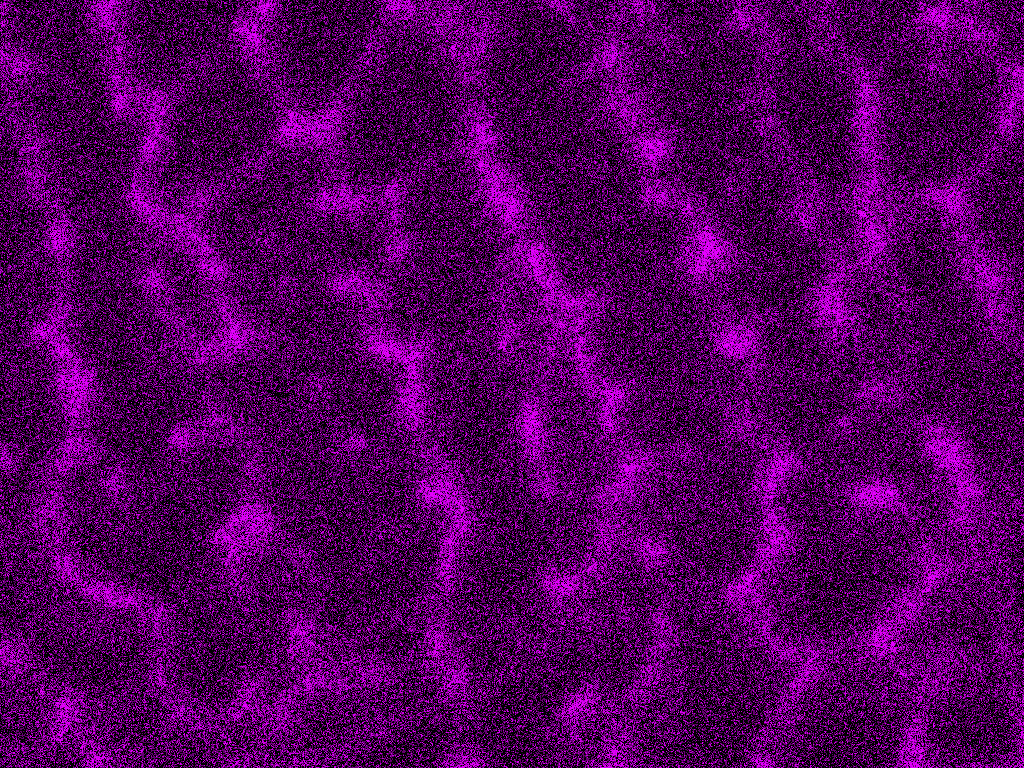

Supplement: Supplementary file 2 [file mmc2.zip › Figure 2 Raw TIFs - Markanday Data-in-Brief/As-DED/Nb L_alpha_1 Map As-DED.tif]

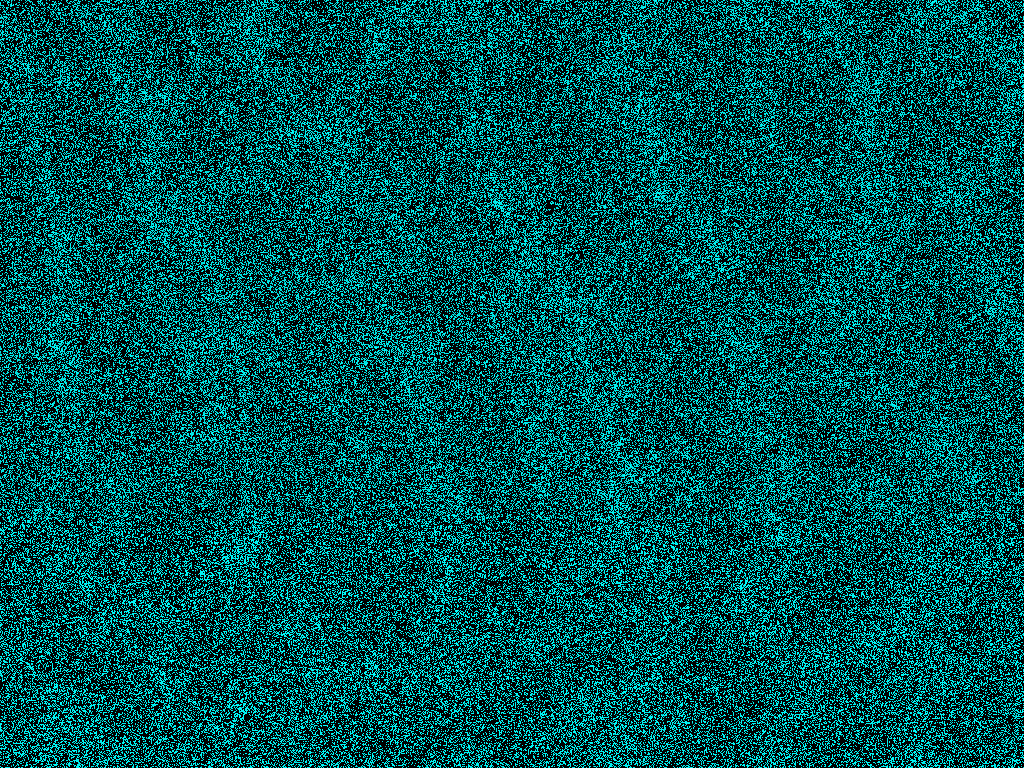

Supplement: Supplementary file 2 [file mmc2.zip › Figure 2 Raw TIFs - Markanday Data-in-Brief/As-DED/Ti K_alpha_1 As-DED.tif]

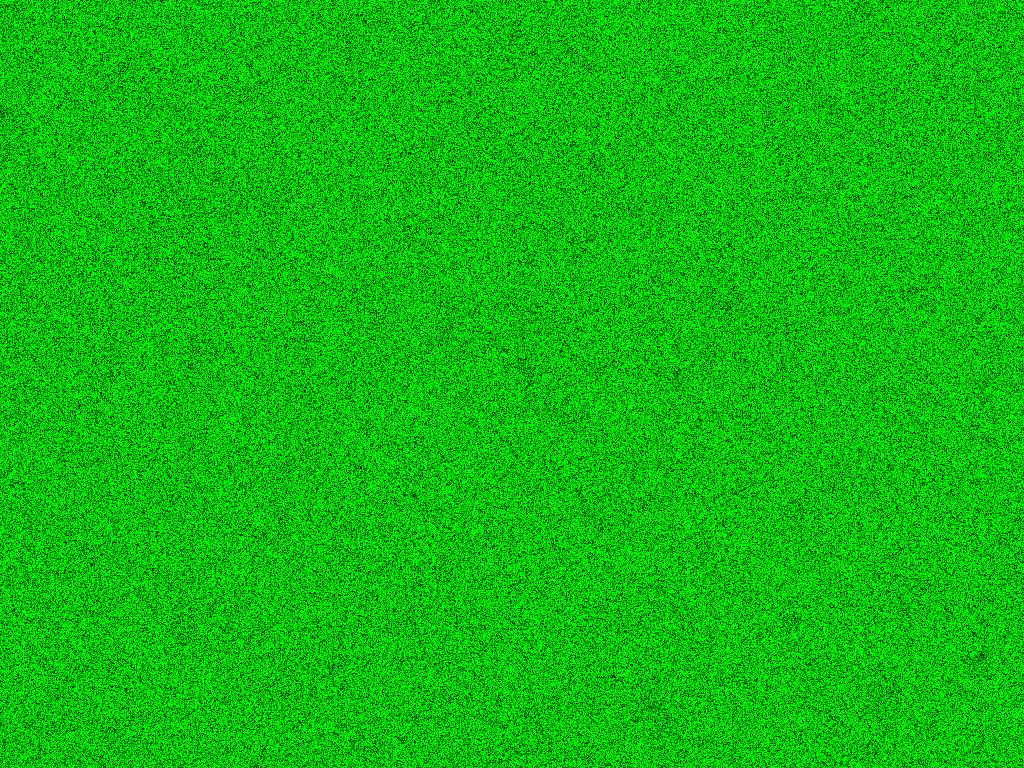

Supplement: Supplementary file 2 [file mmc2.zip › Figure 2 Raw TIFs - Markanday Data-in-Brief/Sample C/Cr K_alpha_1 Map Sample C.tif]

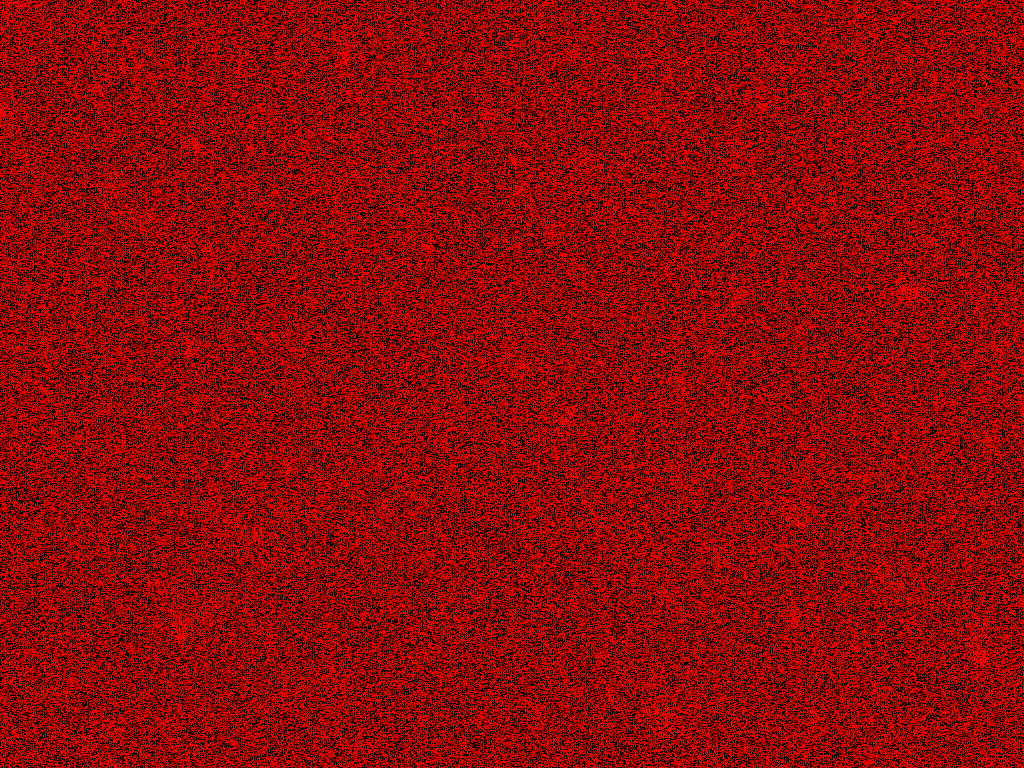

Supplement: Supplementary file 2 [file mmc2.zip › Figure 2 Raw TIFs - Markanday Data-in-Brief/Sample C/Mo L_alpha_1 Map Sample C.tif]

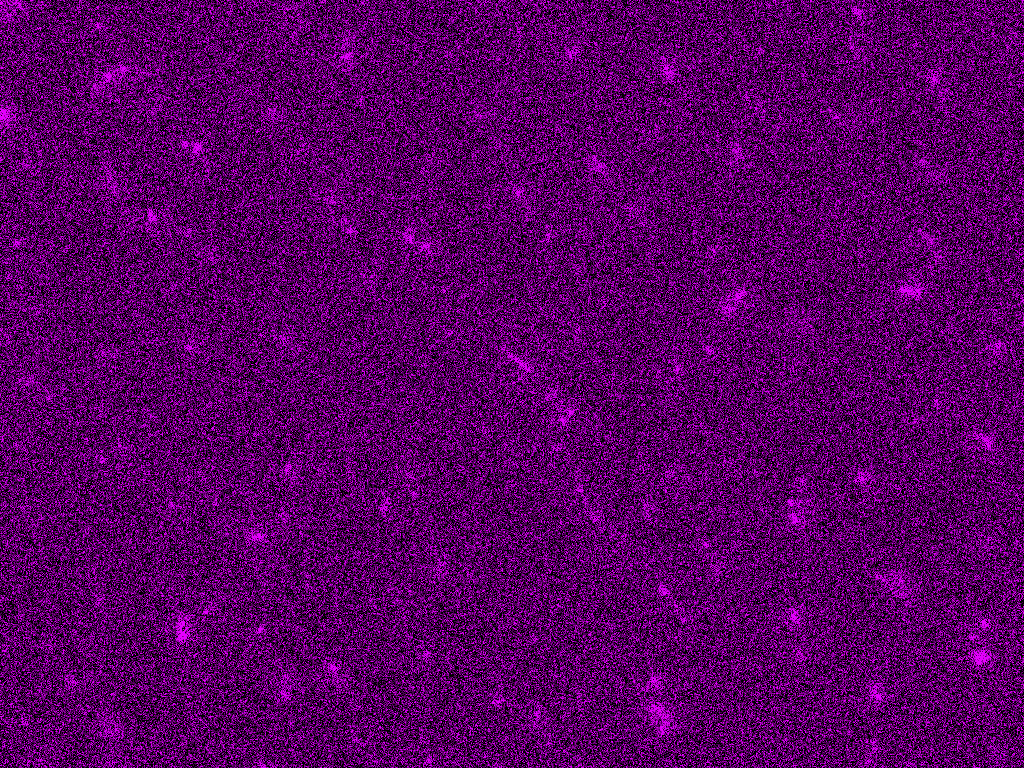

Supplement: Supplementary file 2 [file mmc2.zip › Figure 2 Raw TIFs - Markanday Data-in-Brief/Sample C/Nb L_alpha_1 Map Sample C.tif]

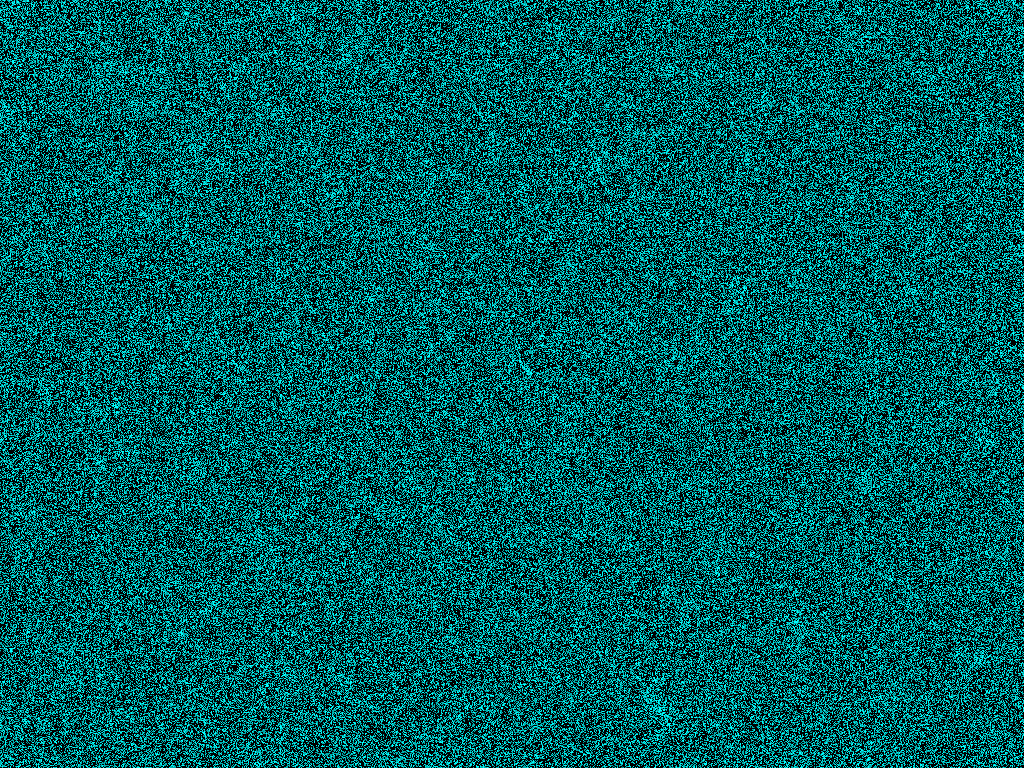

Supplement: Supplementary file 2 [file mmc2.zip › Figure 2 Raw TIFs - Markanday Data-in-Brief/Sample C/Ti K_alpha_1 Map Sample C.tif]

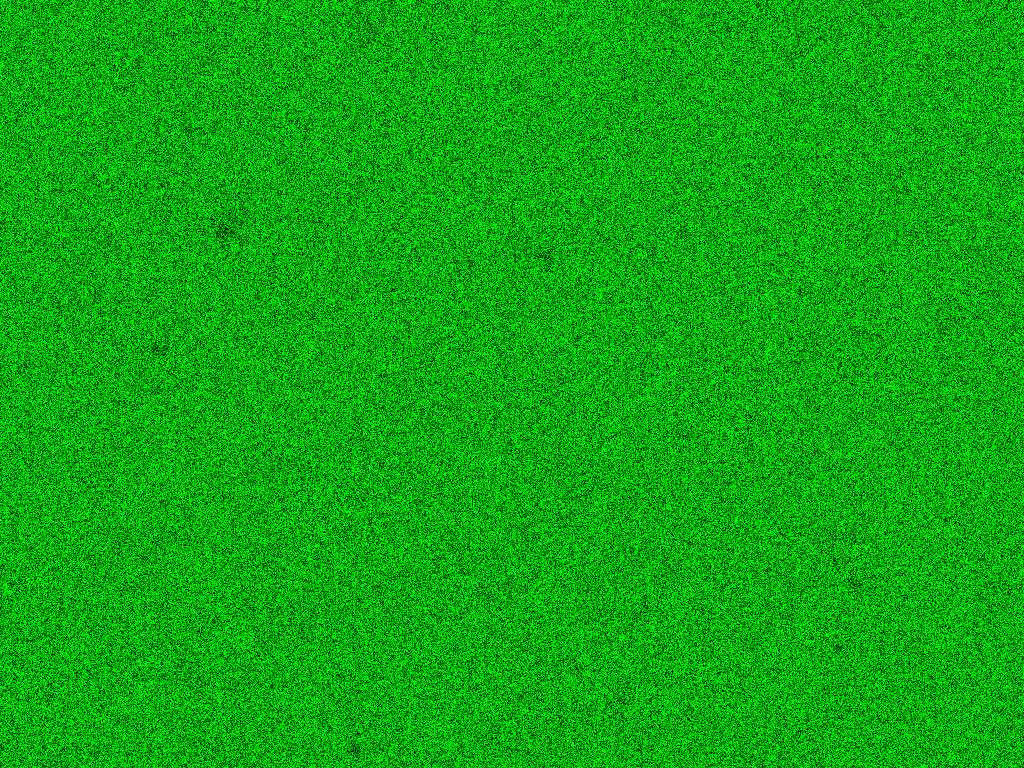

Supplement: Supplementary file 2 [file mmc2.zip › Figure 2 Raw TIFs - Markanday Data-in-Brief/Sample D/Cr K_alpha_1 Map Sample D.tif]

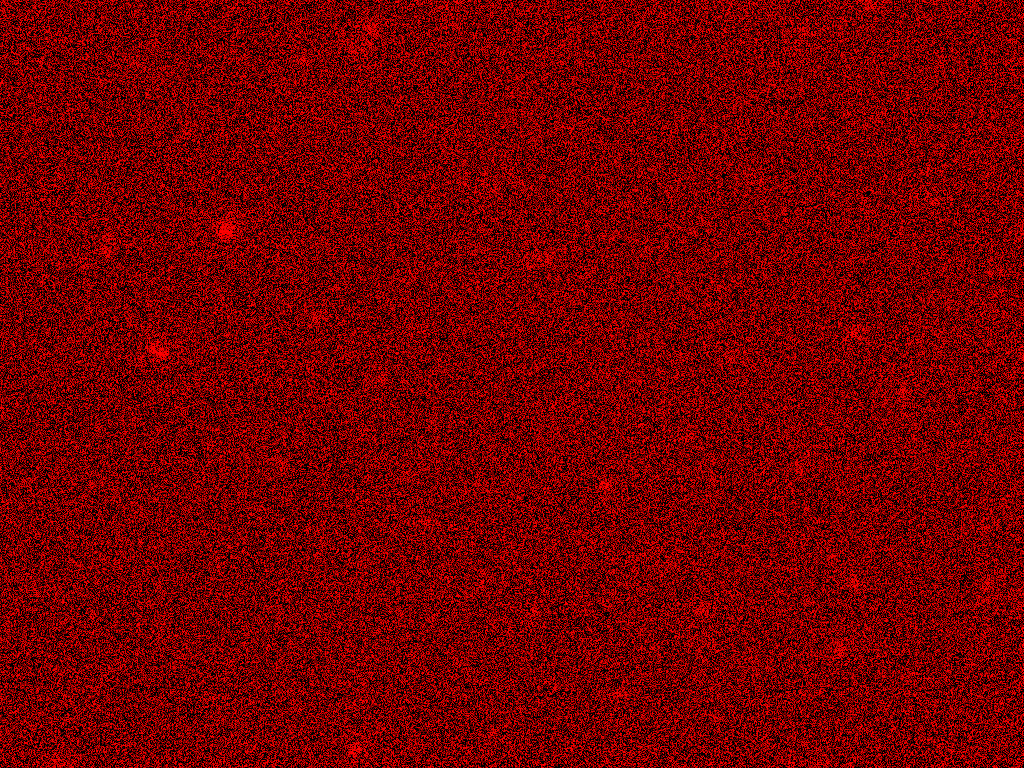

Supplement: Supplementary file 2 [file mmc2.zip › Figure 2 Raw TIFs - Markanday Data-in-Brief/Sample D/Mo L_alpha_1 Map Sample D.tif]

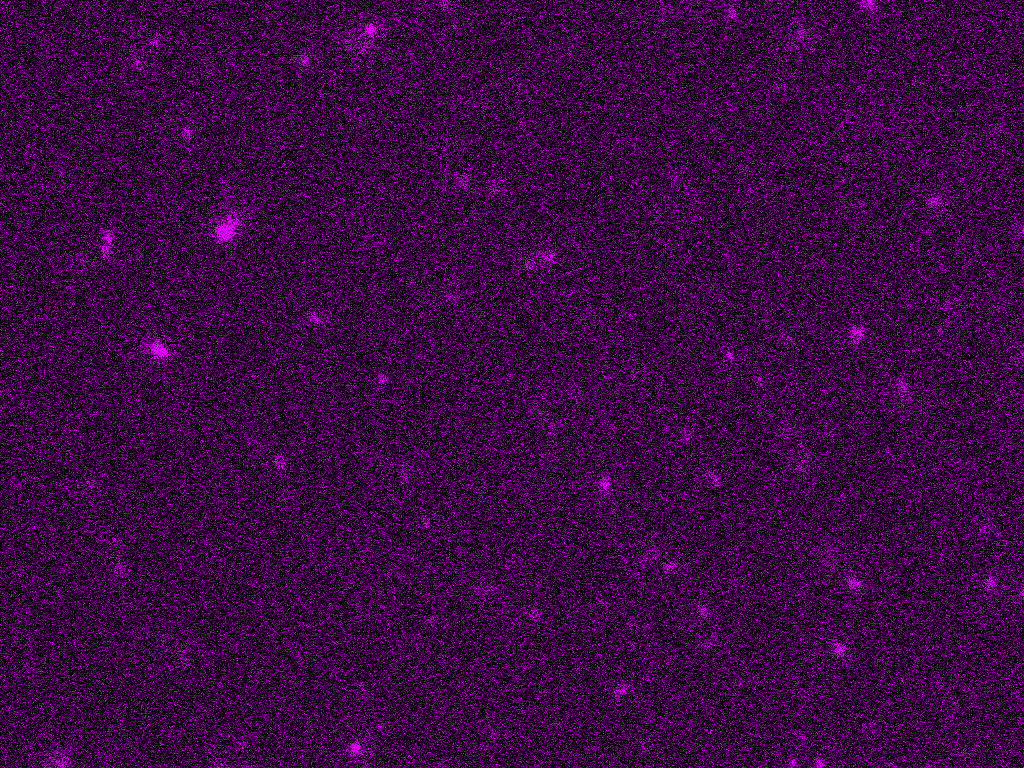

Supplement: Supplementary file 2 [file mmc2.zip › Figure 2 Raw TIFs - Markanday Data-in-Brief/Sample D/Nb L_alpha_1 Map Sample D.tif]

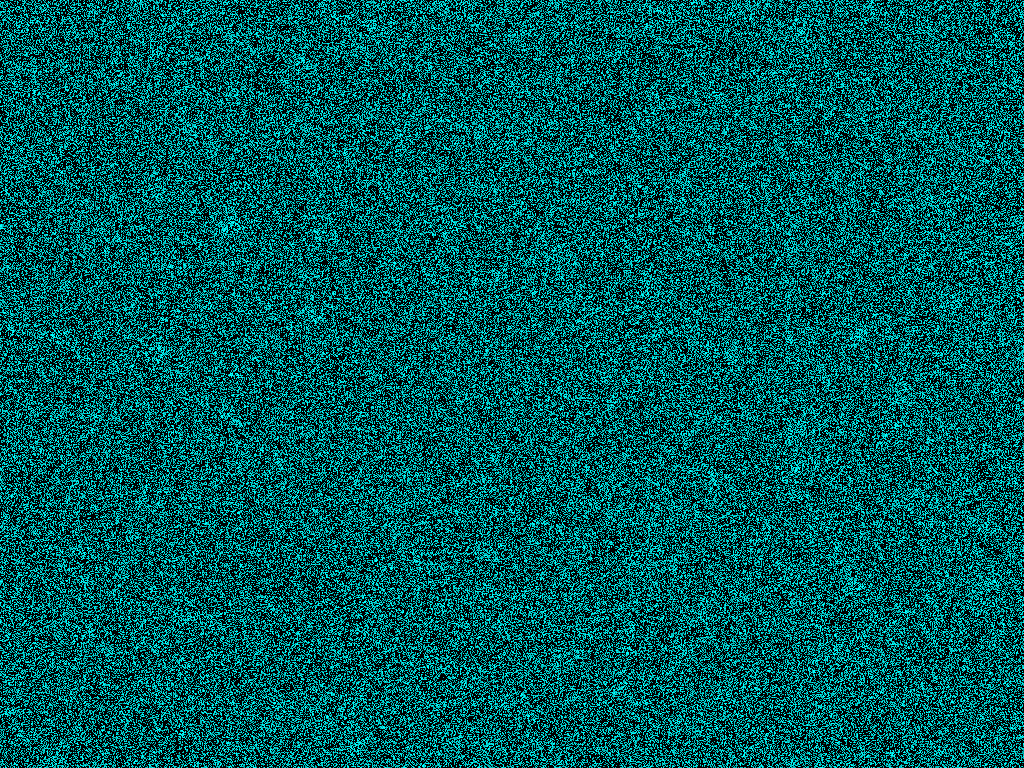

Supplement: Supplementary file 2 [file mmc2.zip › Figure 2 Raw TIFs - Markanday Data-in-Brief/Sample D/Ti K_alpha_1 Map Sample D.tif]
